# Polyarteritis Nodosa With Complications: A Diagnostic Challenge and Management Dilemma

**DOI:** 10.7759/cureus.49677

**Published:** 2023-11-29

**Authors:** Saviz Saghari, Kudret Arslan, Sarah Sordo

**Affiliations:** 1 Internal Medicine, Capital Health Regional Medical Center, Trenton, USA

**Keywords:** cutaneous polyarteritis nodosa, negative skin biopsy, steroid-induced diabetes, vasculitis skin biopsy, polyarteritis nodosa

## Abstract

Polyarteritis nodosa (PAN) is a rare autoimmune vasculitis characterized by the inflammation of medium-sized arteries throughout the body. This case report presents the clinical course of a 48-year-old female patient who experienced a complex diagnostic journey and complications during the management of PAN. The patient initially presented with dry skin, rash, and pruritus, which led to an extensive evaluation. Despite multiple visits and investigations, the definitive diagnosis of PAN was delayed. Eventually, the patient was diagnosed with PAN based on skin biopsy findings demonstrating vasculitis and inflammation of blood vessel walls. The treatment course was further complicated by the development of steroid-induced diabetes and recurrent vasculitis symptoms. Recurrence of symptoms, including rashes and neuropathy, necessitated adjustments in therapeutic interventions. This case highlights the challenges encountered in managing both PAN and its associated complications and emphasizes the importance of a multidisciplinary approach and improved patient compliance to optimize treatment outcomes.

## Introduction

Polyarteritis nodosa (PAN) is a systemic vasculitis characterized by the inflammation of medium-sized arteries. While there is a milder variant of the disease known as cutaneous polyarteritis nodosa (CPAN), PAN primarily involves systemic manifestations [1]. CPAN specifically affects the skin and presents with various characteristic manifestations, including nodules, ulcers, purpura, livedo reticularis, and subcutaneous nodules. Skin lesions associated with PAN often serve as early indicators of underlying systemic involvement [2].

Unlike other medium-vessel vasculitides, PAN is typically not associated with anti-neutrophil cytoplasmic antibodies (ANCA). Even in cases with milder disease activity, there is significant morbidity due to digital ulcerations, ischemia, and uncomfortable skin nodules [3]. Systemic PAN can develop in patients with CPAN, albeit less frequently.

## Case presentation

A 48-year-old female initially presented to our clinic in 2018 with generalized dry skin, rash (blisters on thighs and shoulders), and pruritus, with no improvement observed despite multiple visits and treatment attempts with diphenhydramine, loratadine, and triamcinolone cream. The patient reported a history of similar dry, scaly, and itchy rashes on the extremities, which were biopsied by a dermatologist a few years prior, yielding no significant pathology at that time; therefore, no systemic steroid was prescribed.

**Figure 1:**
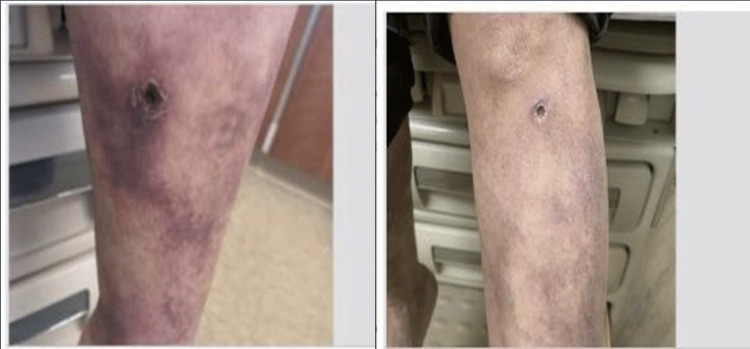
Panel A (right): 1x1 cm ulcer and pruritic rashes on the leg, Panel B (left): 2x2 cm ulcer with irregular edges, worsening rashes with skin discoloration, and edema.

Over the following months, the patient's condition continued to worsen, with recurrent papular pruritic rash appearing on the arms and legs, along with peri-orbital and lower extremity edema with epidermal ulceration (Figure 1, Panel A). Echocardiogram revealed an ejection fraction of 55-60% with no systolic or diastolic dysfunction. Renal workup including serum creatinine, urinalysis, and kidney ultrasound came back unremarkable. Ankle-brachial index (ABI) was also within the normal range. Compression stockings were recommended initially, presuming a diagnosis of stasis dermatitis related to the scaly, pruritic rashes on the legs, but no symptomatic improvement was noted.

**Figure 2:**
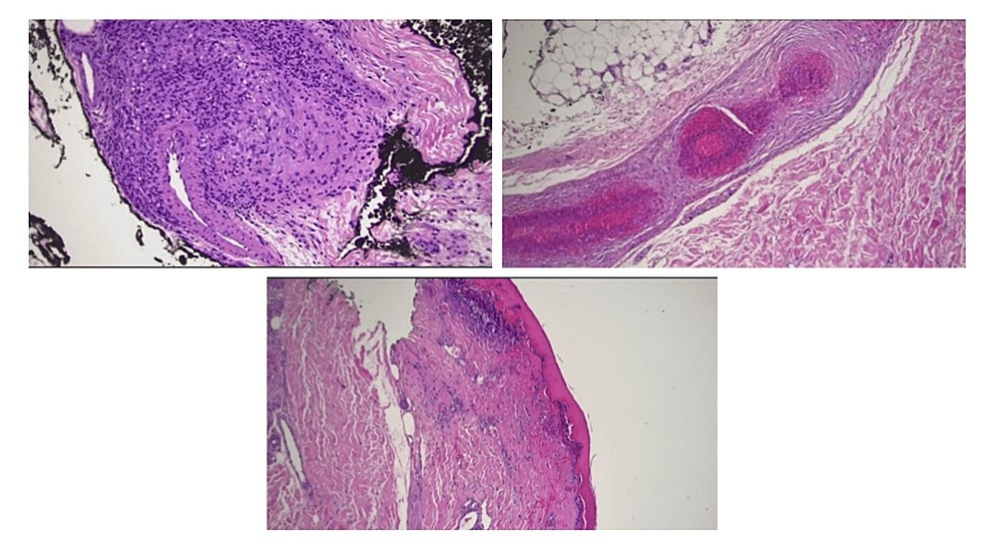
Image 1 (left upper) shows epidermal ulceration. Image 2 (right upper) shows a medium-sized blood vessel in the deep dermis with fibrinoid necrosis, acute and chronic inflammatory cell infiltrates, and intravascular thrombus formation. Image 3 (bottom) shows a blood vessel with chronic inflammatory cell infiltrates.

Subsequently, the patient returned with progressive fatigue, facial edema, and worsening rash of the lower extremity (Figure 1, Panel B). Lyme/human immunodeficiency virus workup yielded negative results, and rheumatology workup (anti-nuclear antibody, rheumatoid factor, complement component 3 (C3), complement component 4 (C4), erythrocyte sedimentation rate, C-reactive protein) as well as ANCA were unremarkable; however, significant eosinophilia of 11.6% was noted (Table 1). A skin biopsy performed in 2019 revealed vasculitis with inflammation of blood vessel walls consistent with PAN (Figure 2) despite negative ANCA testing.

**Table 1:** Laboratory test results IFA, immunofluorescence assay; C-ANCA, cytoplasmic, anti-nuclear cytoplasmic auto-antibody; P-ANCA, peri-nuclear, anti-nuclear cytoplasmic auto-antibody; CRP, C-reactive protein; ESR, erythrocyte sedimentation rate; WBC, white blood cell

No	Lab tests	Value	Reference value
1	Antinuclear antibody, IFA	negative	Borderline: 1:80
2	Complement C4, serum	15 mg/dL	14-44 mg/dL
3	Complement C3, serum	94 mg/dL	82-167 mg/dL
4	C-ANCA	<1:20	Negative: <1:20
5	P-ANCA	<1:20	Negative: <1:20
6	CRP	0.6 mg/dL	<1 mg/dL
7	ESR	2 mm/hour	0-30 mm/hour
8	Eosinophil count	11.6%	<5% of total WBC count

In light of worsening clinical symptoms, the patient was started on systemic steroids. During the course of treatment, the patient developed steroid-induced diabetes as a complication, requiring additional management strategies. She also experienced recurrent vasculitis symptoms, including painful rashes and neuropathy. Despite treatment adjustments and subsequent therapy with mycophenolate mofetil, the patient continued to experience flare-ups.

## Discussion

PAN is a rare form of vasculitis characterized by the inflammation of medium-sized arteries. Around 25% to 40% of patients with PAN get skin lesions as the initial symptom [4]. Serious difficulties in many organs and tissues, including kidneys, gastrointestinal tract, heart, and neurological system, might develop as a result of late detection. While systemic PAN may result in organ damage and neuropathy, which can cause pain and motor impairments, CPAN can produce painful skin nodules and ulcerations. The prognosis for untreated PAN is dismal, with a five-year survival rate of 13% [5]. PAN patients' outcomes have improved with therapy; the five-year survival rate is more than 80% [5]. Diagnosis of PAN can be challenging due to its varied clinical presentation and the absence of specific serological markers such as ANCA. A negative skin biopsy does not definitively rule out PAN [6]. In some cases, the characteristic inflammation may not be evident in the initial biopsy. If clinical suspicion persists despite negative results, repeat biopsies or additional tests may be necessary to confirm the diagnosis accurately [7].

CPAN, a milder variant of PAN primarily affecting the skin, can precede or coexist with systemic PAN. The presence of eosinophilia in our patient's laboratory workup further complicated the diagnostic process [2].

The delayed diagnosis in this case can be attributed to the atypical presentation and the lack of characteristic serological markers, leading to a prolonged diagnostic journey. Cutaneous manifestations, such as dry, scaly rashes and papular pruritic lesions, preceded the development of systemic symptoms. The diagnosis of PAN was ultimately confirmed through a skin biopsy showing vasculitis.

The treatment of PAN typically involves immunomodulating agents to control the systemic inflammation. Glucocorticoids are the first-line treatment, but their long-term use can lead to complications such as steroid-induced diabetes, as observed in our patient [8]. Disease-modifying antirheumatic drugs such as mycophenolate mofetil may be used as steroid-sparing agents or for disease control in refractory cases. However, some patients may still experience disease flare-ups and require adjustments to their therapeutic regimen [9].

## Conclusions

This case report highlights the diagnostic challenges and complications encountered in the management of PAN. The atypical presentation and absence of specific serological markers prolonged the diagnosis, resulting in delays in appropriate management. Additionally, the development of steroid-induced diabetes further complicated the treatment course. A multidisciplinary approach involving dermatologists, rheumatologists, and endocrinologists is crucial for effective management and optimal treatment outcomes. Improved patient compliance and regular follow-up are essential to monitor disease activity, adjust therapeutic interventions, and minimize the risk of complications in patients with PAN.
